# Impact of Sleep and Its Disturbances on Hypothalamo-Pituitary-Adrenal Axis Activity

**DOI:** 10.1155/2010/759234

**Published:** 2010-06-09

**Authors:** Marcella Balbo, Rachel Leproult, Eve Van Cauter

**Affiliations:** Sleep, Chronobiology and Neuroendocrinology Research Laboratory, Department of Medicine, The University of Chicago, Chicago, IL 60637, USA

## Abstract

The daily rhythm of cortisol secretion is relatively stable and primarily under the influence of the circadian clock. Nevertheless, several other factors affect hypothalamo-pituitary-adrenal (HPA) axis activity. Sleep has modest but clearly detectable modulatory effects on HPA axis activity. Sleep onset exerts an inhibitory effect on cortisol secretion while awakenings and sleep offset are accompanied by cortisol stimulation. During waking, an association between cortisol secretory bursts and indices of central arousal has also been detected. Abrupt shifts of the sleep period induce a profound disruption in the daily cortisol rhythm, while sleep deprivation and/or reduced sleep quality seem to result in a modest but functionally important activation of the axis. HPA hyperactivity is clearly associated with metabolic, cognitive and psychiatric disorders and could be involved in the well-documented associations between sleep disturbances and the risk of obesity, diabetes and cognitive dysfunction. Several clinical syndromes, such as insomnia, depression, Cushing's syndrome, sleep disordered breathing (SDB) display HPA hyperactivity, disturbed sleep, psychiatric and metabolic impairments. Further research to delineate the functional links between sleep and HPA axis activity is needed to fully understand the pathophysiology of these syndromes and to develop adequate strategies of prevention and treatment.

## 1. Introduction

The release of nearly every hormone in humans is characterized by daily oscillations that result mainly from the interaction between circadian rhythmicity and the sleep-wake cycle. Circadian rhythms are generated by the endogenous master pacemaker located in the paired suprachiasmatic nucleus (SCN) of the hypothalamus and are kept entrained to the local conditions by environmental timing cues; the most important of these cues is light, that signaled to the SCN by direct input from the retina via the retinohypothalamic tract [1]. In addition to the master clock in the SCN, circadian oscillators have been found in a number of peripheral tissues, such as liver, heart, lung, skeletal muscle, and adrenal glands [2, 3].

Apart from circadian rhythmicity and sleep-wake cycle, plenty of other factors which tend to recur at regular 24-hour intervals, such as postural changes, food intake, exposure to light and dark, have an influence on daily hormonal changes. 

Given the close interaction of these periodic factors, the study of the impact of each single component, and in particular of sleep, on the hormonal profiles has faced several methodological problems: sleep is, indeed, a period of fasting, associated with changes in posture and light exposure. Furthermore, the tight concordance of habitual sleep and wake times with certain circadian phases has made it difficult to distinguish sleep and circadian effects.

Hence, specific protocols have been designed to dissociate the contribution of the alternation of sleep and wake states from that of circadian process on 24-hour rhythms of hormonal release. The most common study protocols involve shifts of the sleep period (with volunteers kept awake at night and allowed to sleep at different times of the day), sleep deprivation, including constant routine conditions (in which human volunteers remain awake for 30–60 hours, in a semirecumbent position, in dim light and receive frequent small identical meals) [4] and ultradian protocols (in which subjects live on a very short “day” such as a 20 or 90 or 180 minutes “day,” with 2/3 of the time awake and 1/3 of the time asleep) [5–7].

## 2. Physiology of Hypothalamo-Pituitary-Adrenal (HPA) Axis

The hypothalamo-pituitary-adrenal (HPA) axis is the major neuroendocrine mediator of the stress response. A stressful stimulus perceived by the senses ultimately induces the release of CRH from the paraventricular nucleus (PVN) of the hypothalamus. CRH stimulates the release of ACTH from the anterior pituitary and ACTH subsequently initiates the liberation of glucocorticoids from the adrenal cortex. Cortisol has numerous actions, including feedback inhibition at the level of the PVN and the anterior pituitary to control CRH or ACTH synthesis and release. In addition, the HPA axis receives relevant feedback from other areas of the brain, such as the hippocampus and amygdala [8]. An important interplay is also the excitatory reciprocal interaction between the HPA axis and the brainstem sympathetic locus coeruleus (LC)-norepinephrine (NE) system: CRH activates the LC; in turn, NE, a known wake-promoting neurotransmitter, activates hypothalamic CRH [9, 10].

**Figure 1:**
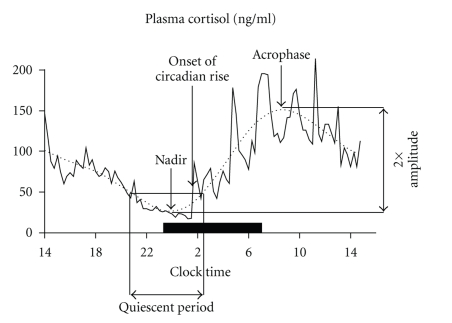
24-hour individual cortisol profile (solid line).The smoothing curve (dotted line) is calculated to determine the minimum (nadir), the maximum (acrophase), the onset of the circadian rise, and the amplitude (50% of the difference between the acrophase and the nadir) of the cortisol profile. The black bar represents the sleep period.

The basal activity of the HPA axis displays a clear daily rhythm: the 24-hour profiles of peripheral levels of ACTH and cortisol occur in close parallelism and are characterized by high levels in the early morning (acrophase), a decline throughout the day, with a prolonged period of low levels (quiescent period), centered around midnight (nadir), and a rapid rise during the second half of the night. The amplitude of the circadian oscillation is generally defined as 50% of the difference between the value of the acrophase and the value of the nadir [11–13] (Figure 1). The nocturnal onset of the cortisol rise and the morning peak may be seen as the response to an endogenous stimulus driven by the circadian clock, as it is suggested by the fact that the onset of the cortisol rise takes many days to adapt to an abrupt shift of the sleep-wake and light-dark cycle [14]. The daylong cortisol decline may represent the rate of recovery of the axis from this endogenous challenge. 

The results of constant routine, shifted sleep, and ultradian schedule experiments have revealed prominent endogenous circadian components in this well-characterized and highly reproducible daily rhythm. As a confirmation, lesions of the SCN in rats result in the loss of corticosteroid periodicity [15, 16]. The SCN shows, indeed, direct and indirect projections (mainly via the subparaventricular zone and the dorsomedial nucleus of the hypothalamus) to the PVN [17, 18]. In addition, significant evidence now points to a neural SCN-adrenal gland connection, via the autonomic nervous system and independent from the HPA axis activation [19, 20]: this multisynaptic pathway from the SCN to the adrenal gland is able to transmit light information to the adrenal glands, modulating corticosterone release without changes in ACTH secretion [21]. Moreover, a role in the circadian regulation of glucocorticoid secretion is probabily played by an intrinsic circadian oscillator in the adrenal glands: indeed, a study by Valenzuela and colleagues has recently demonstrated the rhythmic expression of clock genes in the adrenal cortex of a diurnal primate [22]. Notably, the oscillation of the transcriptional expression of the adrenal clock genes, accompanied by rhythmic expression of the steroidogenic enzyme 3-beta-hydroxysteroid dehydrogenase, was directly modulated by melatonin, shown to be a potent time signal for the entrainment of peripheral circadian oscillators, but was also maintained for 36 hours in cultured adrenal explants, independently from the SCN oscillations. 

The distinct circadian pattern of cortisol secretion may be driven not only by circadian oscillators, but also by metabolic factors. The HPA axis plays a key role in mobilizing energy resources in conditions of reduced energy supply, such as during hypoglycemia [23]. In response to HPA axis activation, both liver and kidney increase endogenous glucose production, ensuring sufficient glucose supply to glucose-dependent organs, in particular the brain [24]. Correspondingly, mild transient hyperglycemia has been shown to suppress HPA secretory activity in rats [25]. In a recent study, nocturnal glucose administration in young healthy males significantly reduced the early morning rise in ACTH and cortisol concentrations, regardless of whether the subjects were asleep or awake, supporting the view that increasing energy demands of the brain toward the end of the night essentially contribute to the early morning peak in the HPA axis activity [26].

The impact of age and sex on HPA function has been an object of great interest and a matter of debate. Most early studies investigating this topic, limited by small sample sizes and heterogeneous composition, seemed to indicate that there are no major sex and age differences in basal functioning of the HPA axis [27–29]. However, aging has been more often associated with an hyperactivation of the axis in response to a stimulus: interestingly, what appears to be more affected is the ability of the axis to recover from a challenge (resiliency), rather than the rate of the initial response or the magnitude of the response [29, 30]. 

**Figure 2:**
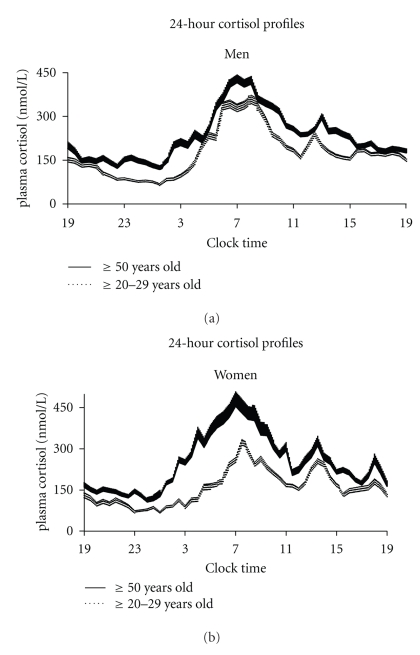
Effects of age and sex on cortisol profile: Mean 24-hour (+ or − SEM) cortisol profiles in men (left profiles) and women (right profiles) for 2 age groups that is, 50 years of age and older (solid lines) and 20 to 29 years old (hatched lines). (adapted from Van Cauter [30])

A study that analyzed almost 200 temporal profiles of plasma cortisol from men and women, with ages from young adulthood to late senescence, found, indeed, subtle but substantial sex and age-related differences [31] (Figure 2). In young adulthood, women appeared to have, compared to men, lower 24-hour mean cortisol levels, a longer quiescent period (that started earlier and ended later), a lower acrophase, and a lower absolute amplitude of the circadian variation. Therefore, the female response to the circadian signal appeared to be slower and of lesser magnitude than in men and the recovery from the morning peak more rapid with a longer quiescent period; a modulatory role of estrogens in the greater resiliency of the HPA axis observed in premenopausal women cannot be ruled out and might be involved in their lower rate of cardiovascular risk compared to men. In the same study, aging was associated, in both sexes, with a progressive increase of 24-hour mean levels from the 2nd to the 8th decade, associated with a shorter quiescent period and higher nadir values. These findings are consistent with the reported association of aging with an impairment in the inhibitory feedback mechanisms and in the ability of the system to turn off the signal after the morning stimulation [29]. A sex difference in the effects of aging on HPA function was apparent as in women, but not in men, an increase in the level of the acrophase was detected, as expression of increased responsiveness of the axis; moreover, the reduction in the duration of the quiescent period appeared to be even sharper in women than in men. Sleep complains in older adults are more common in women than in men and this could play a role in the greater female reduction of the quiescent period. Of note, the most common sleep disorder, obstructive sleep apnea, is as prevalent in older women as in older men. In addition, the timing of the nadir and of the onset of the nocturnal cortisol rise are advanced in older adults of both sexes and the circadian amplitude, when expressed as a percentage of the 24-hour mean level, is decreased: hence, during senescence a progressive advance of circadian phase seems to occur, associated with a loss of strength of the circadian signal.

Another recent protocol investigated the effect of age, gender and BMI on the pulsatile pattern of secretion of ACTH and cortisol [32]. In this study, mean ACTH concentrations, basal and pulsatile ACTH secretion directly correlated with BMI and were higher in men than in women, after controlling of BMI. The secretory regularity of ACTH and cortisol was also evaluated with approximate entropy (ApEn) statistic; the univariate ApEn quantifies the orderliness of a single hormone release, while the bivariate cross-ApEn quantitates the relative pattern synchrony of coupled time series: increased randomness of coupled hormonal secretion patterns is interpreted as reflecting weaker feedback and feedforward control mechanisms [33]. ApEn analysis revealed that greater age is associated with more irregular patterns of cortisol, but not ACTH secretion, suggesting that aging might alter intra-adrenal paracrine or neurogenic mechanisms that modulate glucocorticoid secretion. In addition, in men, ACTH-cortisol feedforward synchrony and cortisol-ACTH feedback synchrony both decline with age, as quantified by cross-ApEn, denoting a deterioration of coordinate control of ACTH and cortisol secretion patterns.

## 3. Effects of Normal Sleep on HPA Axis

Although modest, effects of sleep on cortisol profile are clearly present and have been well documented.

Sleep is a dynamic process characterized by the alternation between two different states: REM and non-REM sleep (NREM). NREM sleep is further classified into 3 different stages of increasing intensity (stage 1, 2, and slow wave sleep, or SWS). Each sleep stage has characteristic EEG frequencies and waveforms. During REM sleep the EEG resembles that of stage 1, with relatively low amplitude, mixed frequency waveforms; muscle tone is inhibited and eye movement are present. NREM sleep includes a variably synchronous cortical EEG: in particular SWS exhibits high-amplitude delta frequency (0.5–4.5 Hz) oscillations as a defining feature and are considered to have a fundamental role in the restoration of cognitive, emotional, and metabolic functions.

Some changes in cortisol profile seem to be sleep-state dependent, others may be related to specific sleep stages.

### 3.1. Impact of Sleep Onset and Offset

Sleep onset exerts a modest inhibitory effect on cortisol profile that persists for 1-2 hours. This was evident in subjects undergoing a 3-hour sleep-wake cycle [34], in subjects reversing the phase of sleep-wake cycle by 180° [35] and during temporal isolation (free running condition) [36]. Under free-running conditions, sleep is generally initiated at a much later phase of the endogenous circadian cortisol rhythm, that is, after the rising phase of cortisol has begun; in this case sleep onset interrupts the rise of cortisol and inhibits its secretion for 1–3 hours. In a similar way, sleep onset during habitual wake time lowers cortisol levels [37]. In a study that compared the effect of nighttime sleep (sleep period 2300–0700) with daytime sleep (sleep period 0700–1500, that is, sleep onset at the peak of corticotropic activity), a transient decrease in cortisol levels at the time of sleep onset was observed [13].

An influence of sleep-wake transitions on cortisol secretion is also detectable during nocturnal awakenings which are consistently associated with a transient elevation of cortisol levels, followed by temporary inhibition of cortisol secretion [38].

Similarly, the final morning awakening elicits a rapid and marked rise in cortisol levels, augmenting the circadian acrophase of cortisol concentrations, but in contrast to nocturnal awakenings, the elevation of cortisol levels associated with final morning awakening persists for about one hour. In addition, it is detectable independently of the time and the mode of awakening (naturally or induced) [39], both in long and short sleepers [40]. In the previously mentioned work from Weibel et al. the acrophase of cortisol occurred at the same time in the morning both in nighttime sleepers and in daytime sleepers, demonstrating the strength of circadian rhythmicity, but the amplitude of the morning pulse was smaller if the subjects were asleep [13].

### 3.2. Modulatory Effects of Sleep Stages

A consistent temporal association between low cortisol levels and high SWS has been demonstrated [41]. The inhibitory effect that sleep onset seems to exert on HPA axis has been mostly attributed to SWS. Indeed the quiescent period of HPA activity, although starting before sleep onset, occurs during the first half of the night when SWS is present to its greatest degree [34, 35] and some authors have even hypothesized the existence of an unknown factor, possibly GHRH secreted during SWS, exerting inhibitory properties on corticotropic activity [42]. Cortisol levels have been shown to decrease in the first 20 minutes after the onset of SWS [41]; in addition, a study that compared morning sleep after a regular night of sleep, with morning recovery sleep after a night of sleep deprivation, found increased SWS and decreased cortisol levels in the latter condition [43], in agreement with the hypothesis of an inhibitory role of SWS on cortisol secretion. Furthermore, the response of ACTH and cortisol to a stimulus (CRH administration, alone or in association with vasopressin) has been shown to be blunted if the test was performed during nighttime sleep, and in particular during SWS, compared with nighttime wakefulness [44, 45].

Nevertheless, some authors have questioned the direction of the inverse link found between cortisol and SWS, suggesting that a decreased HPA tone may promote sleep deepening and, conversely, that increasing cortisol levels may prevent the occurrence of SWS [13, 46]. Gronfier et al, investigated the relationship between cortisol secretion and slow-wave activity (SWA), the spectral power in the low frequency range of the EEG (0.5–4.5 Hz), that is considered a stable trait dependent marker of the intensity of SWS. These authors showed that, during the period of pulsatile release, the cortisol secretory variations were concomitant with or anticipated opposite variations in SWA, by 10–20 minutes [47], suggesting the existence of a common central control.

An association between REM sleep and periods of low adrenal cortex secretory activity has been reported [48] and it has been shown that a significant proportion of REM sleep phases starts at a time of stable or decreasing cortisol levels, but may persist after cortisol increases again [41]. In a study investigating simultaneous chronological changes in sleep duration and quality and in the 24-hour cortisol profiles from young adulthood to old age, it was observed that a decrease in the amount of REM sleep (stable and unchanged until 50 years of age, decreased by approximately 50% in late life compared with young adulthood) occurred in synchrony with an elevation of evening cortisol levels. A trend for an association between lower amounts of REM sleep and higher evening cortisol concentrations, independently of age, was also detected [49].

The association between cortisol secretion and specific sleep stages has also been investigated in relation to memory consolidation. It is well known that a central cognitive function of sleep is to consolidate newly acquired memories for long-term storage [50] and, moreover, different sleep stages seem to be implicated in the consolidation of different types of memory [51]. Retention of declarative memory critically depends on the integrity of the hippocampus and has been shown to benefit specifically from early nocturnal sleep, in which SWS predominates and cortisol concentrations are minimal [52, 53]. Consistent with a role for quiescent cortisol release in this process, hippocampus-dependent declarative memory has been impaired by the infusion of a low dose of cortisol during a period of early SWS-rich sleep [54]. A similar result has been achieved with the administration of the glucocorticoid receptor (GR) agonist dexamethasone [55]. Thus, inhibition of cortisol secretion and in particular inactivation of GRs appear to be critical for hippocampus-mediated formation of declarative memories.

When declarative memory for highly emotional rather than neutral material is assessed, the data point to a more beneficial influence of REM sleep which prevails during the late night, when cortisol concentrations rise [56]. Declarative memory for emotional events, although still hippocampus-dependent, is under critical modulation by the amygdala [57]. A recent randomized, double-blind, placebo-controlled study examined the effect of the cortisol synthesis inhibitor metyrapone on sleep-associated consolidation of memory for texts of either neutral and emotional valence [58]. Metyrapone suppressed cortisol concentrations, especially the rise in cortisol during late sleep, and decreased time spent in SWS, which was replaced by shallow NREM sleep. REM sleep was preserved. While memory for the neutral texts was reduced, interestingly metyrapone even amplified emotional enhancement in text recall, which has been shown in many clinical and neuroimaging studies to depend on the amygdala [59–61]. Thus, the late night rise in cortisol appears to dampen amygdala-dependent emotional processing and could exert a protective function by preventing excessive emotionality in memory. 

Cortisol secretory pattern, in association with specific EEG frequencies, has been studied not only during sleep, but also during wake: during daytime wakefulness cortisol release follows the increase in EEG absolute power of the beta frequency band (13–35 Hz) with a 10-minute delay [62]; this finding has been subsequently confirmed in a more recent study [63] in which a significant temporal association has been found between cortisol secretory rate and waking EEG absolute power in the gamma frequency range (20–45 Hz), in a group of young males not sleep deprived. Given that high-frequency EEG over 20 Hz is considered a marker of daytime arousal [64], a functional link between the regulatory mechanisms controlling the HPA activity and the level of central arousal has been hypothesized.

## 4. Effects of Shifted Sleep on Hpa Axis

As mentioned before, one of the strategies to differentiate between effects of circadian rhythmicity and effects of sleep-wake homeostasis on physiological variables has been to design studies of shifted sleep, in which volunteers are submitted to a large sudden advance or delay in their dark-light, rest-activity, sleep-wake cycle, similar to what occurs in jet lag or shift work rotations. Hence, these protocols allow for the effects of circadian modulation to be observed in the absence of sleep and for the effects of sleep to be observed at an abnormal circadian time. The most important external time cue, that aligns the circadian system with the environment, is the light-dark cycle, and these experiments take advantage of the fact that the circadian pacemaker takes several days to adjust to an abrupt shift in the dark-light cycle. The rate of re-entrainment of the biological rhythms to the new environmental time has been recognized to be different among physiologic variables, being slower for those mainly controlled by the circadian process, and faster for those mainly under the sleep-wake homeostasis control [65]. In the cortisol profile, the end of the quiescent period and the onset of the daily rise are mostly under the control of circadian rhythmicity, while the timing of the acrophase and the amplitude of the diurnal variation are more influenced by sleep-wake transitions. Indeed, in studies of real and simulated jet-lag, the synchronization of the acrophase to the new clocktime occurred faster than the adaptation of the quiescent period [14, 66]. 

### 4.1. Delay of the Sleep-Wake Cycle

**Figure 3:**
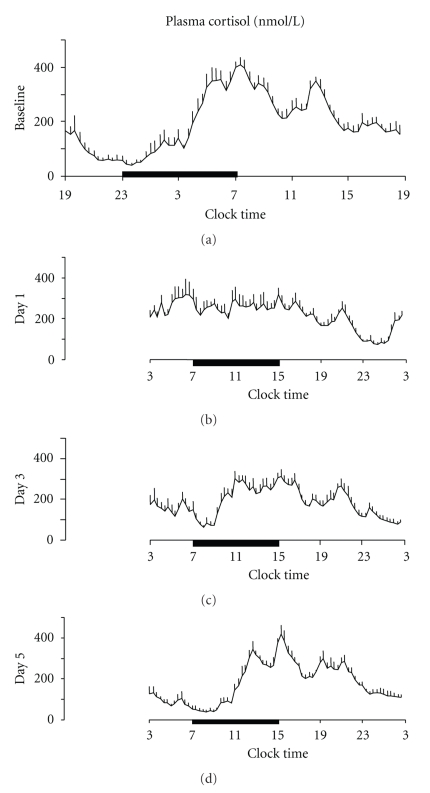
Effect of an 8-hour sleep delay on cortisol profiles. Mean 24-hour (+ SEM) cortisol profiles under baseline conditions (bedtimes from 23:00 to 7:00), and one, three, five days after an 8-hour delay of the sleep-wake and dark-light cycles (bedtimes from 7:00 to 15:00). Black bars represent the sleep periods. (Adapted from Buxton et al. [67].)

The effect of an abrupt 8-hour delay of the sleep-wake cycle on plasma cortisol profiles was investigated in a study designed to mimic westward travel across eight time zones [67]. The protocol involved a three-day period of habituation in which the subjects followed a standardized schedule with fixed meal times and sleep time (23:00–7:00). On day 4, baseline 24 hours hormonal profiles starting at 18:00 and sleep recordings were obtained. At the end of the baseline sampling period, the subjects were kept awake until 7:00 the next day, and then allowed to sleep between 7:00 and 15:00, that is, 8 hours later than under baseline conditions. Nocturnal wakefulness was enforced in light intensity averaging 1.500 lux, that is, very bright indoor light. For the next four 24-hour spans the same sleep schedule was maintained. Blood samples for 24-hour hormonal profiles, starting at 2:00, and sleep recordings were collected on the first, third, and fifth days of the shifted sleep period. Cortisol profiles are shown in Figure 3.

On the first day after the shift, profound disruptions in the 24-hour cortisol rhythm were found: a reduction of more than 3 hours in the duration of the quiescent period resulted in an increase of the 24-hour mean levels and in the advance of the circadian onset. Both a higher nadir value, due to the lack of the inhibitory effect of sleep onset, and a lower acrophase value, due to the lack of the stimulatory effect of awakening, were observed. As a consequence the relative amplitude of the rhythm was reduced by approximately 40%. The timing of the acrophase was delayed by about 3 hours and 30 minutes. On the third day, as a sign of partial adaptation to the new schedule, the circadian onset of cortisol was delayed by more than 5 hours, while the timing of the acrophase was delayed by more than 6 hours. Relative amplitude remained lower than at baseline. On day 5, the cortisol profile had essentially been adapted to the new schedule.

### 4.2. Advance of the Sleep-Wake Cycle

**Figure 4:**
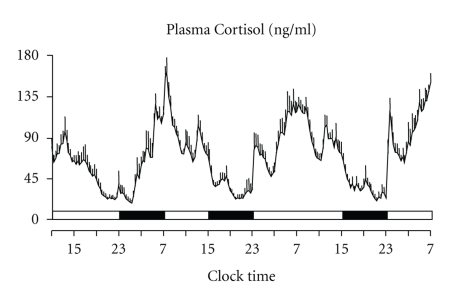
Effect of an 8-hour sleep advance on cortisol profiles. Mean (+ SEM) cortisol profiles obtained during 68 consecutive hours including baseline conditions and 2 postshift periods, that is, after an 8-hour advance of the sleep-wake and dark-light cycles. Black bars represent the sleep periods whereas wake time was spent in dim light conditions. (Adapted from Caufriez et al. [68].)

The impact on HPA axis activity of an acute 8-hour advance of the rest-activity cycle has also been examined [68]. In this study, eight healthy young subjects were studied for four consecutive days. The first day was a baseline condition and sleep was allowed between 23:00 and 7:00; on day 2 the sleep period was advanced, and subjects were allowed to sleep between 15:00 and 23:00. The same shifted cycle was maintained on day 3. Blood samples for plasma cortisol were collected for 68 consecutive hours, starting at 11:00, before the baseline night (Figure 4). In the first postshift period, compared with the baseline profile, the timing of the nadir was advanced by about 3 hours and 20 minutes; the quiescent period was markedly reduced mainly due to an advance of the offset by more than 3 hours. No shift in the timing of the acrophase occurred, thus the duration of the rising phase of cortisol secretion was increased by nearly 3 hours and presented a biphasic pattern. No significant changes were found in the acrophase value. These changes, together with the slight increase in the nadir values, resulted in a trend toward higher 24-hour mean levels. During the second postshift period (day 3), minor signs of further adaptation of the cortisol profiles were detected: there was no further advance of the timing of the nadir and only a minor additional advance of the onset of the quiescent period. However a further shift of almost 1 hour was observed for the offset of the quiescent period. Hence, the findings of this study showed that an abrupt 8-hour advance of the sleep-wake, rest-activity and dark-light cycle results in a 3- to 4-hour advance of the timing of the nadir and of the end of the quiescent period, which are both reliable markers of central circadian rhythmicity. In this protocol, however, there was a temporal coincidence between the phase markers and the sleep-wake and dark-light transition (at 23:00), which elicited robust cortisol pulses. Thus, the advance of the timing of the nadir and of the end of the quiescent period may only partly reflect an advance of the circadian phase, because it could also be partly due to the effect of the awakening from the shifted sleep. The lack of adaptation of the timing of the acrophase to the new schedule has been interpreted as a consequence of the biphasic pattern of the cortisol rise after the shift: the first peak induced by the sleep-awakening transition may have delayed the subsequent circadian rise and prevented the advance of the timing of acrophase. Therefore, despite a relatively fast adaptation of some circadian markers, the lack of adaptation of acrophase, the longer duration of the rising phase, and the shorter duration of the quiescent period resulted in elevated cortisol levels at the time of the usual trough of the profile. Circadian misalignment may lead to central and peripheral deleterious consequences, such as memory deficit and insulin resistance [69–71].

## 5. Effects of Sleep Loss and Sleep Disrupion on HPA Axis

Given the modest but clearly detectable inhibitory effect exerted by sleep onset on the activity of the HPA axis, it has been hypothesized that sleep loss might increase cortisol levels. Protocols examining both acute total sleep deprivation (prolonged wakefulness for 24 hours, 48 hours, or even 72 hours) and semichronic partial sleep restriction (reduction of the nocturnal time in bed for a variable number of days) have been designed to test this hypothesis. 

### 5.1. Acute Total Sleep Loss

Several studies have reported an elevation of cortisol levels both during the night of total sleep deprivation [13, 35, 72] and, if wakefulness was further prolonged, during the following day, particularly in the afternoon and evening [63, 73]. The findings of high glucocorticoid levels in a condition of acute sleep loss have been interpreted as reflecting the stress of the effort to maintain wakefulness and is consistent with the positive correlation found between high frequency EEG activity in wake, indices of arousal, and cortisol release [63].

However, divergent findings have also been reported. Indeed, some authors have reported no change [41, 43, 74, 75] or even a slight decrease in cortisol levels [76, 77] after one or several nights of total sleep deprivation. These discrepancies could be due to a lack of control of experimental conditions, such as physical activity and posture, and/or to an insufficient frequency of blood sampling; indeed, short naps may have gone undetected and estimation of cortisol levels with infrequent blood sampling are often inaccurate. Findings of decreased cortisol levels after sleep deprivation have been interpreted as related to an increase in fatigue and sleepiness. Notably, the decrease in plasma or urinary cortisol found by Kant et al. [77] and Åkerstedt et al. [76] occurred after 48 hours or more of prolonged wakefulness. Hence, a biphasic HPA response to sleep deprivation has been suggested [63] where activation of the HPA axis, as part of the stress response, may be one of the arousal mechanisms recruited early on to adapt to sleep loss; however, when wakefulness is prolonged, the increased sleep pressure may eventually cause a blunting of the HPA axis activity.

Some protocols have examined cortisol profiles during the recovery night after a night of total sleep deprivation. Again contradictory results have been reported: while in some studies cortisol secretion seemed to be almost unaffected by recovery sleep following prolonged wakefulness [78–80], Vgontzas et al. [81] found a significant decrease in plasma cortisol levels, that appeared to be negatively correlated with the rebound increase of SWS. The timing of the period of recovery sleep is likely to have played a role in these conflicting findings.

### 5.2. Semichronic Partial Sleep Restriction

Recurrent partial sleep restriction has become a widespread habit in modern society [82] and its hormonal and metabolic consequences have only begun to be appreciated. 

**Figure 5:**
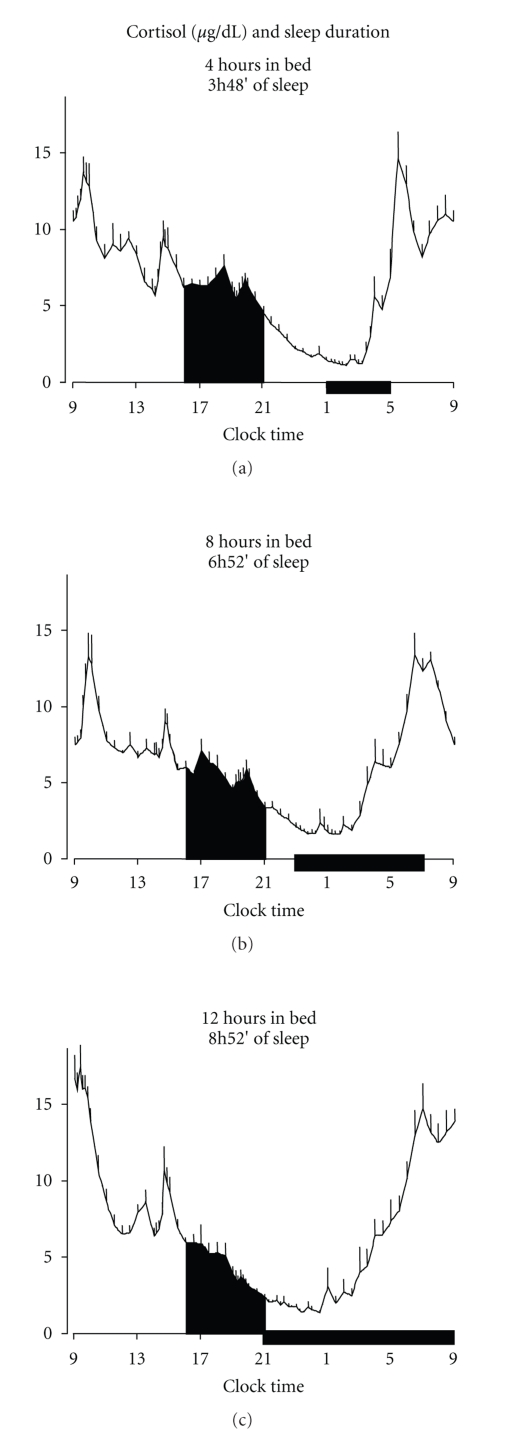
Inverse “dose-response” relationship between evening cortisol levels and sleep duration. Mean 24-hour (+ SEM) cortisol profiles and AUC (black areas) under 4 hours, 8 hours, and 12 hours bedtimes. Black bars represent the sleep periods. (Adapted from Spiegel et al. [84].)

The first systematic study of HPA axis activity in a state of sleep debt assessed the effect of 6 consecutive nights of 4 hours in bed in eleven young men [83]. The sleep debt condition was compared with a fully rested condition, obtained after 6 nights of 12 hours in bed. Plasma and salivary cortisol were measured under both conditions. The state of sleep debt, as compared to the fully rested state, was associated with elevated cortisol concentrations in the afternoon and in the early evening and with a shorter quiescent period, due to a delay in its onset by nearly 1.5 hour. In addition, the rate of decrease of free cortisol concentrations in saliva between 16.00 hours and 21.00 hours was about six times slower in the sleep-debt than in the fully rested condition. Nine of the eleven subjects of the previous study participated, one year later, in a separate protocol with 8-hour bedtime, using the same experimental procedures. Interestingly, cortisol evening levels observed under 8-hour bedtime condition were intermediate between those measured under 4-hour and 12-hour bedtime conditions [84] (Figure 5).

Consistent findings indicative of an elevation of evening cortisol levels following partial sleep loss had been previously reported after only 1 night of sleep restricted to 4 hours in bed [73]. In this study, plasma cortisol levels over the 1800–2300 hour period were 37% higher on the day following the night of restricted sleep than on the previous day, and the onset of the quiescent period occurred 1 hour later.

Thus, sleep deprivation appears to be associated with an elevation in evening cortisol levels that may reflect decreased efficacy of the negative feedback regulation of the HPA axis. Even if modest, this elevation may result, under conditions of chronic sleep loss, in a significant glucocorticoid overload and consequently expose the body to the central and peripheral deleterious effects associated with glucocorticoid excess. 

In this regard, it is notable that chronic short sleepers have been found to have higher nocturnal cortisol levels compared with chronic long sleepers [40], suggesting that, even in the long term, mechanisms of adaptation and down-regulation of the HPA axis fail to occur. In a cross-sectional analysis of data collected from the Whitehall II study, the relationship between self-reported sleep duration, sleep disturbances, and salivary cortisol levels has been assessed in a cohort of more than 2700 middle-aged men and women [85]. Short sleep duration was associated with an acute increased cortisol awakening response and both short sleep duration and disturbances were independently associated with a slower rate of decline of cortisol levels across the day and thus with increased evening levels.

An increasing number of epidemiological studies have reported an association between short sleep duration and higher risk of developing obesity and type II diabetes [86–89]. The evidence summarized above suggests that HPA axis hyperactivity could be one of the mechanisms involved in the pathogenesis of these metabolic impairments.

### 5.3. Impact of Disrupted Sleep Quality

Only a few experimental studies have examined the effect of changes in sleep quality on activity of the HPA axis. Some of them were designed to suppress specific stages of sleep, others involved sleep fragmentation across all stages of sleep.

The two studies that examined the impact of SWS suppression on the temporal profiles of cortisol secretion had negative findings: the earlier study [90] conducted in young men, compared the nocturnal cortisol profile during a baseline night with that observed during partial SWS sleep suppression (between 2300 and 0200). The more recent study [91] investigated 24 hours cortisol profiles in a group of young men and women after 2 nights of normal sleep and after 3 nights of selective suppression of SWS throughout the night. In both studies, suppression of SWS was achieved by sending acoustic stimuli to the bedside whenever delta waves occurred and both studies attempted to avoid complete awakenings. Therefore stages of deep sleep were replaced with more shallow sleep, without increase in wake after sleep onset. No significant changes in cortisol profiles were found.

Decreased cortisol levels have been reported in a study of REM suppression [92]. However, REM sleep suppression was achieved through brief awakenings, resulting in a significant increase in wake time.

A recent experiment of sleep fragmentation led to different findings [93]. Following 2 nights of experimental sleep fragmentation across all stages, an increase in morning cortisol levels was observed, in association with a shift in the sympatho-vagal balance, estimated from the analysis of the heart rate variability towards an increase of sympathetic nervous system activity, and a decrease in insulin sensitivity.

## 6. HPA Axis and Sleep Disturbances in Clinical Syndromes

While sleep loss and sleep disruption are generally thought of as activating the HPA axis, there is in turn strong evidence that elevated CRH tone increases sleep EEG frequency, thereby decreasing SWS, and increasing light sleep and wakefulness [94]. In addition, CRH reciprocally activates the locus coeruleus/norepinephrine (LC/NE) system, one of the most important components of the arousal systems [95]. Consistently, CRH1 receptors, mediators of the CRH action, have been extensively studied as potential drug targets and a large number of CRH1 receptors antagonists have been developed for the treatment of stress-related diseases as well as sleep disorders [96]. Glucocorticoids, on the contrary, may exert a dual effect on sleep, depending on the prevailing concentrations: low levels of cortisol are associated with mineralocorticoid receptor (MRs) binding in the hippocampus, that mediate a negative feedback on hypothalamic CRH secretion. In contrast, at higher cortisol levels, glucocorticoid receptors (GRs) are activated, which may exert either inhibitory or excitatory feedback on CRH, depending on the location of the receptors. In times of stress, amygdala GRs may be preferentially activated, increasing CRH secretion, therefore promoting sleep disruption [8]. Moreover, it is well known that activation of HPA axis plays an essential causative role in the pathogenesis of metabolic and mood disorders that are often also associated with sleep disturbances [97, 98]. 

Given these functional links, it is not surprising that HPA hyperactivity, disturbed sleep, psychiatric disorders, and metabolic impairment are characteristic features of several clinical syndromes. We therefore describe below the interactions between sleep and HPA activity in insomnia, depression, Cushing's syndrome, and sleep disordered breathing (SDB).

### 6.1. Insomnia

Insomnia is a sleep disorder characterized by difficulties falling or staying asleep or having restorative sleep, associated with daytime impairment or distress [99]. Even though it is one of the most commonly encountered sleep disorders in medical practice, the understanding of its neurobiological basis and etiology is still limited and therefore even the treatment of this condition is challenging and often unsatisfactory [100].

Despite the evidence linking sleep and HPA axis, and the clear association between insomnia and perceived stress [101, 102], of which the HPA axis is a major mediator, there is a paucity of studies assessing HPA axis activity in this disorder. 

**Figure 6:**
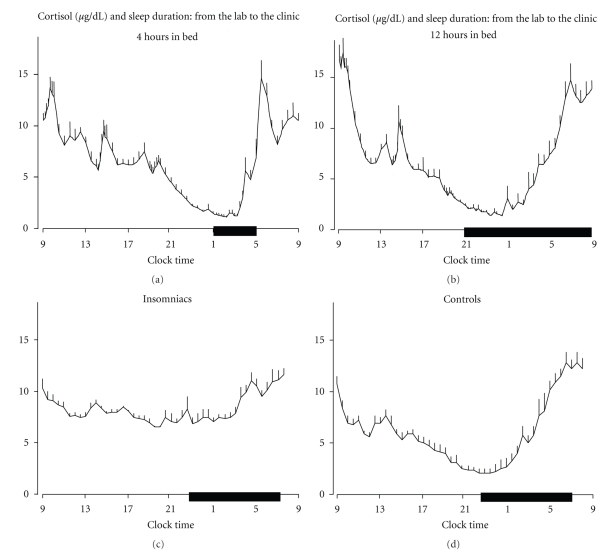
Cortisol rhythm under different sleep conditions. (a) and (b): 24-hour (+ SEM) cortisol profiles under 4 hours (a) and 12 hours (b) bedtimes (Adapted from Spiegel et al. [84]). (c) and (d): 24-hour (+ SEM) cortisol profiles in young insomniacs (c) and age, and BMI-matched controls (d) (Adapted from Vgontzas et al. [105]). Black bars represent the sleep periods.

An early study failed to show any difference in urinary cortisol excretion between control and self-reported poor sleepers [103]. However, the EEG recorded difference between the two groups was only half an hour in total sleep time: such a small difference may have prevented the detection of alteration in cortisol secretion. On the contrary, in a more recent experiment, 24-hour urinary free cortisol excretion was positively correlated with polysomnographic indices of sleep disturbance in a group of adult insomniacs [104]. Moreover, a study that collected 24-hour plasma ACTH and cortisol profiles in young insomniacs and in matched healthy controls found mean ACTH and cortisol levels higher in insomniacs than in controls, with the stronger elevations observed in the afternoon, in the late evening and in the early part of the night [105] (Figure 6). In addition, within the insomniacs, those with a high degree of sleep disturbance, compared with those with a low degree of sleep disturbance, secreted a higher amount of cortisol, particularly in the evening and in the nighttime periods. 

It is still matter of debate if the activation of the HPA system observed in insomnia is secondary to sleep loss or is, on the contrary, a marker of increased central CRH activity. In the latter case, the elevation of CRH tone, possibly following the repeated exposure to a stress, such as in the post traumatic stress disorder (PTSD), may be primarily responsible for the sleep disturbance. Even though some authors lean towards this second hypothesis, suggesting an HPA axis dysfunction as causative factor in the pathogenesis of insomnia [8, 105], a contribution of sleep loss to the evening cortisol elevation seen in insomniacs, cannot be ruled out. An intriguing and plausible model has been proposed to explain the perpetuation of chronic insomnia. In fact, evening cortisol levels while awake correlate with the number of awakenings during the subsequent night in subjects with and without insomnia [106, 107]. If elevated HPA activity before sleep promotes sleep fragmentation, sleep fragmentation and sleep loss have in turn been shown to increase evening cortisol levels, as discussed before. Taken together, these data suggest the occurrence of a vicious circle that could be responsible for the chronicity of insomnia. Of note, multiple studies have shown that insomnia precedes and is a risk factor for the development of psychiatric conditions, including depression and anxiety [101]. The activation of HPA axis in chronic persistent insomnia might play a role in the pathogenesis of these disorders.

### 6.2. Major Depression

Alterations of sleep architecture and HPA axis are commonly observed in patients with major depression. 

Characteristic sleep-EEG findings in depressed patients include disturbed sleep continuity, increased wakefulness, shorter REM latency, and increased REM density [108–110]. Spectral analysis further reveals lower slow-wave activity during the first non-REM period [111]. 

Well-documented neuroendocrine changes in depressed patients include increased cortisol levels, in particular at the time of the cortisol nadir [112, 113], increased ACTH levels [113], lack of inhibition of cortisol to the suppression test with dexamethasone [114, 115] or exaggerated response of ACTH and cortisol to the combined dexametasone+CRH test [116]. An alteration of circadian rhythmicity, namely, a phase advance of the ACTH and cortisol nadir has been found in some studies [117, 118], but not in others [119, 120].

It is still unclear if these neuroendocrine and EEG alterations are a trait marker of depression, detectable independent from the phase (relapse or remission) of the syndrome or a state marker, able, therefore, to normalize during periods of remission.

One report documented a normalization of both ACTH-cortisol profiles and REM sleep in adult patients after recovery [117], but in other studies, cortisol levels or the response to Dex-CRH test were improved after recovery, but pathological sleep-EEG persisted [121–123].

Interestingly, it has been hypothesized that the nature and the extent of the alterations in HPA axis activity and in sleep architecture may help to identify distinct subtypes within major depression [124]. Indeed, an HPA overdrive seems to be most consistently observed in patients with melancholic features, associated with major sleep EEG alterations (low amount of SWS, short REM latency, high REM density). In contrast patients with atypical features may be characterized by reduced HPA activity, hypersomnia and less consistent alterations of SWS and REM sleep [124, 125].

### 6.3. Cushing's Syndrome

Endogenous Cushing's syndrome results from chronic exposure to excess glucocorticoids produced by the adrenal cortex. It may be caused by an excess of ACTH production (usually by pituitary adenomas, less frequently by extrapituitary ACTH- or CRH-secreting tumors) or it can result from the excess secretion of cortisol by unilateral adrenocortical tumors (benign or malignant) or by bilateral adrenal hyperplasia or dysplasia [126].

Hallmarks of the Cushing's syndrome are the loss of the typical circadian rhythm of cortisol secretion [127] associated with the peripheral and central consequences of the glucocorticoid excess: metabolic impairments (hyperinsulinism and insulin resistance decreased glucose tolerance, visceral adiposity, hyperlipidemia, and hypertension), coagulopathy, osteoporosis, and increased risk of psychiatric disorders (major depression, anxiety, mania, and cognitive dysfunctions).

Only a few studies have assessed sleep by polysomnography (PSG) in Cushing's syndrome. 

In a recent experiment that analyzed nocturnal cortisol secretion in a group of patients with pituitary Cushing's syndrome compared with a group of matched healthy subjects, adrenal secretory activity was shown to start predominantly during periods of NREM sleep in both groups. Thus, even though the normal nocturnal circadian oscillation of pituitary-adrenal activity is absent or markedly blunted in Cushing patients, a link between pituitary-adrenal activity and the ultradian rhythmicity of REM-non REM sleep appears to be preserved [127].

Alterations of PSG recordings have been consistently found in Cushing's syndrome, confirming the deleterious effects of glucocorticoids at high concentrations on sleep architecture. A reduction of SWS has been reported [46, 128]. In one study disturbances of sleep continuity (increased sleep latency, enhanced waketime) and of REM sleep (shortened REM latency, elevated REM density) have been found [128]. Interestingly, these sleep alterations are similar to those occurring in major depression.

### 6.4. Sleep Disordered Breathing

Sleep disordered breathing (SDB) and, in particular, obstructive sleep apnea (OSA) are increasingly common conditions characterized by repetitive episodes of hypopnea or apnea, due to the loss of muscle tone in the upper airways; subsequent progressive hypoxemia and hypercapnia occur, followed by autonomic and EEG arousal aimed to open the airway and resume normal breathing. However, as sleep occurs again, the cycle repeats itself, throughout the night. 

Major sleep disturbances result from this condition when untreated: total sleep time and sleep efficiency are reduced and sleep is more fragmented; wake, stage 1 and stage 2 are increased, SWS and REM sleep are markedly reduced [129, 130]. Treatment with continuous positive airway pressure (CPAP), that represents at the moment the most efficacious therapy for sleep apnea, has been shown to improve sleep architecture in SDB patients [131].

Moreover, important metabolic impairments are part of the clinical features that characterize this syndrome: indeed, SDB is associated with elevated body mass index (BMI), hypertension, increased risk of diabetes, insulin resistance and dyslipidemia [132]. Even though, until recently, it was unclear if alterations in glucose metabolism and hypertension were the results of obesity or of SDB per se, there is a growing evidence to indicate that SDB may represent an independent risk factor in the pathogenesis of these metabolic alterations [133–136]. 

The mechanisms that underlie this increased metabolic risk are not fully understood yet. It is likely that an increase in the sympathetic activity, secondary to the respiratory distress [137] or to the sleep loss/fragmentation per se [91] plays a role in the development of metabolic morbidities associated with SDB.

The role of HPA axis has also been investigated, with the hypothesis of a hyperactivation of the axis, due to sleep fragmentation and sleep loss. An additional activating factor could be hypoxia, able to stimulate ACTH and cortisol secretion both in animals and in humans [138–140].

Contradictory findings have been reported regarding the effects of SDB on the HPA system and the results are limited by small sample sizes and methodological differences among the studies, such as variable hours of sleep recording and variable severity of SDB.

Elevated cortisol levels have been found in patients with OSA present in some studies [141, 142] but not in others [143, 144]. Responsiveness of ACTH to CRH administration has been shown to be higher in obese patients without OSA compared with lean subjects and to be even higher in obese patients with OSA [145], probably as the result of an alteration in the central control of ACTH secretion, as well as of an impaired sensitivity to the negative feedback action of glucocorticoids. In a recent study the existence of a mild hypercortisolism in OSA patients has been suggested by the finding of a blunted response of cortisol to the dexamethasone suppression test [146].

The effects of CPAP therapy on cortisol levels have also been investigated. Some authors have reported that CPAP does not reduce cortisol levels [147] or that acute withdrawal of CPAP treatment does not result in an increase in cortisol levels [148]. On the contrary, CPAP does reverse hypercortisolemia according to other authors [149, 150], particularly with prolonged use [142]. Noteworthy, several of these studies were limited in that cortisol was measured at a single time point, and consequently do not measure potential clinically important HPA axis and rhythm changes. Variable compliance levels may also explain discrepant findings. In a recent study that measured nocturnal cortisol profiles, sleep apnea in obese men compared with nonapneic obese controls was associated with an elevation of cortisol levels, which was corrected after 3 months of CPAP use [151].

HPA hyperactivation, as result of sleep disturbance, hypoxemia,s and autonomic activation, may play a significant role in the development of the metabolic alterations that characterize OSA. Given the impact of OSA on sleep architecture, it may contribute to the perpetuation of the sleep disorder. Moreover, it may be a risk factor for insomnia and depression, as both are associated with hypercortisolemia.

## 7. Conclusions

The interaction between sleep and the HPA axis is complex and bidirectional. HPA hyperactivity and decreased sleep duration/quality seem to be tightly linked in a vicious circle and could play an essential causative role in the pathogenesis of metabolic and mood disorders. Hypercortisolism, sleep disturbances, metabolic and psychiatric impairments are common features of several clinical syndromes, such as insomnia, depression, Cushing's syndrome, and SDB. The exact understanding of the interaction between sleep physiology and HPA activity appears to be a crucial prerequisite to better elucidate the physiopathology of these syndromes and may lead to new forms of prevention and treatment.
